# *In silico* identification of *Drosophila melanogaster* genes encoding RNA polymerase subunits

**DOI:** 10.17912/micropub.biology.000320

**Published:** 2020-10-20

**Authors:** Steven J Marygold, Nazif Alic, David S Gilmour, Savraj S Grewal

**Affiliations:** 1 FlyBase, Department of Physiology, Development and Neuroscience, University of Cambridge, Cambridge, U.K.; 2 Institute of Healthy Ageing and the Research Department of Genetics, Evolution, and Environment, University College London, London, U.K.; 3 Pennsylvania State University, Center for Eukaryotic Gene Regulation, University Park, PA, U.S.A.; 4 Clark H Smith Brain Tumour Centre, Arnie Charbonneau Cancer Institute, & Department of Biochemistry and Molecular Biology, University of Calgary, Alberta, Canada

**Table 1. Genes encoding RNA polymerase subunits of Drosophila melanogaster:**
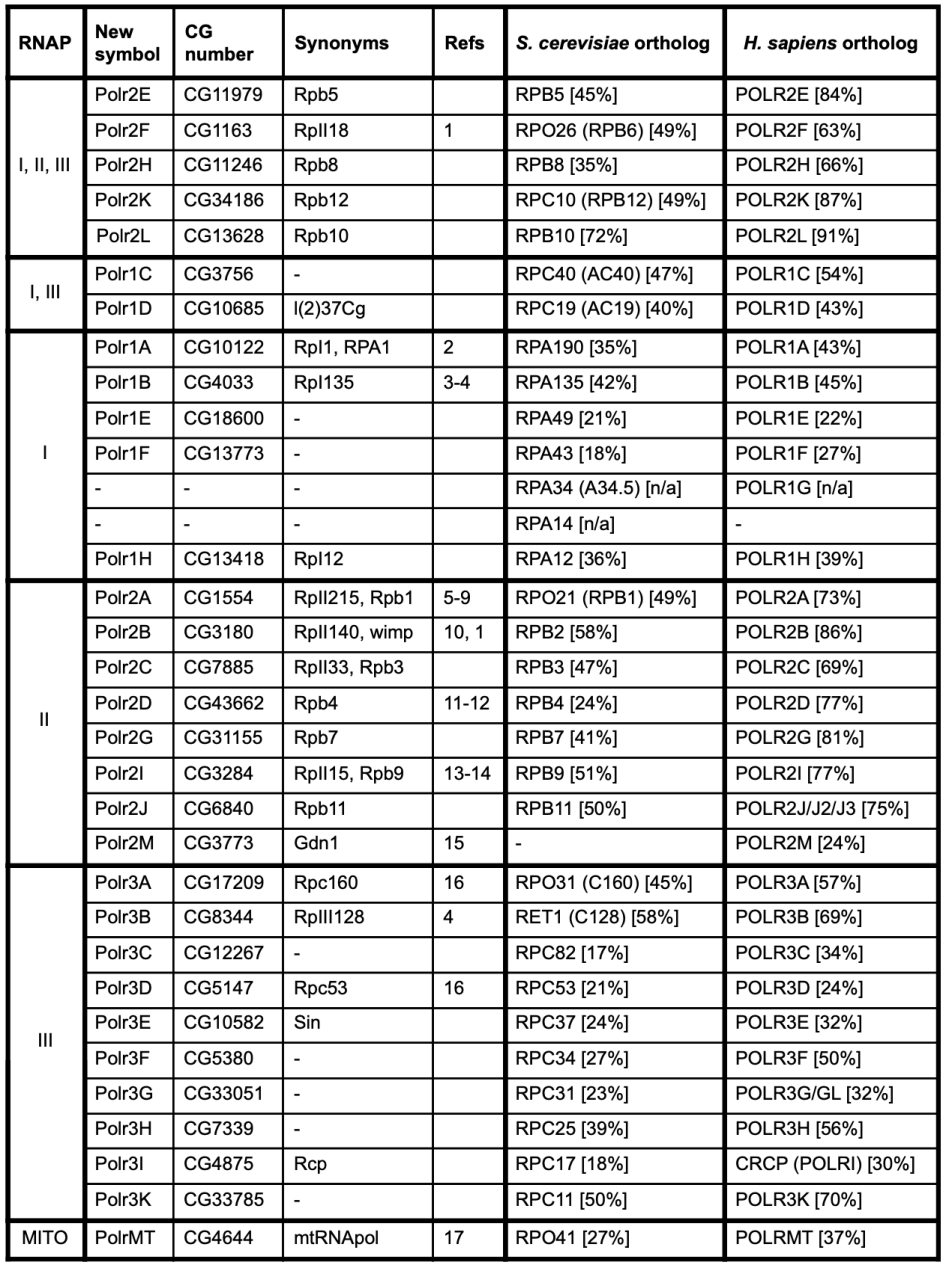
RNAP: RNA polymerase to which the subunit encoded by each gene belongs; New symbol: proposed symbol for the *Drosophila* gene; CG number: gene model annotation ID; Synonyms: notable synonyms/previous symbols of the *Drosophila* gene; Refs: reference(s) identifying/characterizing the *Drosophila* gene/protein with respect to its RNAP function: 1) Hamilton *et al.*. 1993, 2) Knackmuss *et al.*. 1997, 3) Kontermann *et al.*. 1989, 4) Seifarth *et al.*. 1991, 5) Greenleaf *et al.*. 1980, 6) Searles *et al.*. 1982, 7) Greenleaf 1983, 8) Biggs *et al.*. 1985, 9) Jokerst *et al.*. 1989, 10) Falkenburg *et al.*. 1987, 11) Muratoglu *et al.*. 2003 , 12) Pankotai *et al.*. 2010, 13) Harrison *et al.*. 1992 , 14) Liu *et al.*. 1993, 15) Jishage *et al.*. 2018, 16) Filer *et al.*. 2017, 17) Fernández-Moreno *et al.*. 2009; *S. cerevisiae*/*H. sapiens* ortholog: the yeast/human ortholog of the *Drosophila* gene, with the percentage amino acid identity between the encoded proteins given in square brackets (highest identity given if multiple orthologs/isoforms). Note that aligning POLR2A/RPB1 sequences without their C-terminal domain repeat regions does not alter their % identity appreciably (data not shown). Yeast and human symbols reflect official nomenclature used at SGD (Cherry *et al.*. 2012) and the HGNC (Braschi *et al.*. 2019), respectively, with popular alternative nomenclature (e.g. Griesenbeck *et al.*. 2017) given in round brackets.

## Description

Three highly conserved, multisubunit RNA polymerase (RNAP) enzymes, RNAPs I, II, and III, transcribe the eukaryotic nuclear genome (reviewed by Cramer *et al.*. 2008, Vannini and Cramer 2012, Griesenbeck *et al.*. 2017, Cramer 2019). Each one synthesizes different classes of RNA from DNA templates: RNAP I synthesizes the ribosomal RNA precursor that is processed into most ribosomal RNAs (rRNAs), RNAP II makes messenger RNAs (mRNAs) and a variety of non-coding RNAs, and RNAP III synthesizes short, non-coding RNAs including transfer RNAs (tRNAs), the small 5S rRNA and the U6 small nuclear RNA. Each RNAP contains between 12–17 subunits, ten of which form a structurally conserved catalytic core with additional subunits located on the periphery. Notably, five subunits are shared among all three RNAPs and two others are shared between RNAPs I and III. In contrast to the nuclear RNAPs, a single subunit mitochondrial RNAP transcribes the rRNAs, mRNAs and tRNAs of the mitochondrial genome (Arnold *et al.*. 2012).

While much of what we know about eukaryotic RNAP composition and function comes from studies on yeast and human cells, several *Drosophila melanogaster* (hereafter, *Drosophila*) RNAP subunits have also been isolated and characterized, particularly by Greenleaf, Bautz and colleagues in the 1980s–90s (Greenleaf *et al.*. 1980, Searles *et al.*. 1982, Greenleaf 1983, Biggs *et al.*. 1985, Falkenburg *et al.*. 1987, Jokerst *et al.*. 1989, Kontermann *et al.*. 1989, Seifarth *et al.*. 1991, Hamilton *et al.*. 1993, Liu *et al.*. 1993, Knackmuss *et al.*. 1997). Since the publication of the *Drosophila* genome sequence in 2000, the genes encoding all the subunits of RNAP II (Aoyagi and Wassarman, 2000), several subunits of RNAPs I and III (see supplementary data of Filer *et al.*. 2017 and Martinez Corrales *et al.*. 2020) and the mitochondrial RNAP (Fernández-Moreno *et al.*. 2009) have been identified. Nevertheless, a systematic and complete survey of *Drosophila* RNAP genes is lacking, which has resulted in haphazard nomenclature within the fly literature and FlyBase (flybase.org, Thurmond *et al.*. 2019).

We employed a multi-pronged approach to systematically identify all genes encoding *Drosophila* RNAP subunits (see Methods for details). First, we obtained complete lists of RNAP subunits for yeast (*Saccharomyces cerevisiae*) and humans from recent publications and online resources, and used these to identify the *Drosophila* orthologs. Second, we obtained a list of all *Drosophila* genes annotated with relevant Gene Ontology (GO) terms. Importantly, these annotations include those based on direct experimental evidence as well as inferences based on sequence similarity/orthology and the presence of defined protein domains. Finally, we searched the *Drosophila* literature for reports of individual, or lists of, RNAP subunits. The results of these three approaches were cross-checked and integrated, and the results are presented in Table 1.

We find that a total of 31 distinct genes encode RNAP subunits in *Drosophila*. We identified genes encoding the five subunits shared between RNAPs I, II and III as well as the two subunits shared by RNAPs I and III. We also identified genes encoding an additional five subunits of RNAP I, an additional eight subunits of RNAP II, an additional ten subunits of RNAP III and the mitochondrial RNAP. Thus, *Drosophila* possesses twelve RNAP I subunits, thirteen RNAP II subunits, seventeen RNAP III subunits, and a single mitochondrial RNAP. Only a third of these have been characterized directly in *Drosophila*, either biochemically or genetically, with research having focussed on RNAP II subunits and the largest subunits of RNAPs I and III (see Refs column of Table 1). The *Drosophila* subunits show a range of 17–72% (mean of 39%) and 22–91% (mean of 55%) amino acid identity to their orthologs in *S. cerevisiae* and humans, respectively. A comparison of the complement of RNAP subunits across those three species reveals four notable differences: (i) *Drosophila* lacks an identifiable ortholog of yeast RPA34/human POLR1G (Martínez Corrales *et al.*. 2020); (ii) neither *Drosophila* or humans have an ortholog of yeast RPA14 (Russell and Zomerdijk 2006; Martínez Corrales *et al.*. 2020); (iii) yeast lack the POLR2M subunit, which defines a metazoan-specific RNAP II subpopulation (Hu *et al.*. 2006); and (iv) humans possess multiple copies of genes encoding RPB11/POLR2J and RPB7/POLR3G, whereas these are single-copy genes in *Drosophila* and yeast.

Prior to this study, 22 of the 31 *Drosophila* RNAP genes had been named in FlyBase using a variety of conventions. Seven were named based on the empirically determined molecular weight of the *Drosophila* proteins (*RpII18*, *RpI1*, *RpI135*, *RpII215*, *RpII140*, *RpII15*, *RpIII128*), following a nomenclature originally proposed in Greenleaf *et al.*. 1980. Fourteen RNAP genes had been named after their yeast or human ortholog, and one additional gene (*Sin*) was named for an unrelated physical interaction (Dong and Bell, 1999). The remaining nine genes were unnamed or had only a ‘placeholder’ symbol. We wished to assign an informative, systematic nomenclature to all *Drosophila* RNAP genes. Unfortunately, a universal eukaryotic RNAP nomenclature system does not exist, with two different systems currently in use for yeast and humans/vertebrates (Table 1). We propose that the human nomenclature system is adopted for the *Drosophila* genes in FlyBase for the following reasons: (i) individual *Drosophila* RNAP subunits show greater identity to the human subunits compared to yeast; (ii) the overall complement of *Drosophila* RNAP subunits is more similar to humans than yeast; (iii) unlike the yeast nomenclature, the human nomenclature follows a systematic format for all subunits; (iv) using the human nomenclature for the *Drosophila* subunits will facilitate the use/comparison of *Drosophila* data in biomedicine. (The yeast nomenclature will be retained/added to the *Drosophila* gene reports as searchable and browsable synonyms.)

In conclusion, our complete and rationalized listing of *Drosophila* RNAP subunits will be useful to *Drosophila* researchers working in this field as well as to those wishing to compare RNAP biology between fly, yeast, human and other species.

## Methods

Publications identifying/characterizing *Drosophila* RNAP subunits were identified using PubMed (pubmed.ncbi.nlm.nih.gov), FlyBase (flybase.org, Thurmond *et al.*. 2019) and Google (www.google.com). Published lists of *S. cerevisiae* and human RNAP subunits were obtained from Huang and Maraia 2001, Hu e*t al.* 2002, Russell and Zomerdijk 2006, Cramer *et al.*. 2008, Vannini and Cramer 2012 and Griesenbeck *et al.*. 2017. In addition, a curated list of human RNAP subunits was obtained from the HGNC (www.genenames.org/data/genegroup/#!/group/726, Braschi *et al.*. 2019). Ortholog predictions and protein identity percentages were obtained from the integrative ortholog prediction tool, DIOPT (v8) (Hu *et al.*. 2011) via FlyBase. All reported orthologs in Table 1 are reciprocal best hits, with the exception of human POLR3G and POLR3G paralogs, where all genes are listed. Orthology predictions were verified using the HCOP tool (Eyre *et al.*. 2007). The Alliance of Genome Resources database (www.alliancegenome.org (release 3.1.1), The Alliance of Genome Resources Consortium, 2020) was used to query fly, yeast and human for relevant GO annotations (using terms RNA polymerase I complex (GO:0005736), RNA polymerase II, core complex (GO:0005665), RNA polymerase III complex (GO:0005666) and mitochondrial DNA-directed RNA polymerase complex (GO:0034245)). Gene symbol information was obtained from FlyBase (FB2020_03), SGD (www.yeastgenome.org, accessed 17th August 2020) and HGNC (www.genenames.org, accessed 17th August 2020).
